# Teacher Emotional Support and Adolescent Student Burnout: A Moderated Mediation Model of Family Cohesion and Meaning in Life

**DOI:** 10.3390/bs16060955

**Published:** 2026-06-10

**Authors:** Peng Li, Lifang Fan, Xintao Wen, Meng Guo, Wenbin Feng, Ye Wang

**Affiliations:** 1 Faculty of Psychology, Tianjin Normal University, Tianjin 300387, China; lipeng@tjnu.edu.cn (P.L.); fanlifang906@163.com (L.F.); wenxintao2022@163.com (X.W.); gm15840045486_m@163.com (M.G.); fwbpsy@tjnu.edu.cn (W.F.); 2 School of Management, Tianjin Normal University, Tianjin 300387, China

**Keywords:** teacher emotional support, family cohesion, meaning in life, student burnout

## Abstract

(1) Background: Student burnout, widely regarded as a form of “hidden dropout” among adolescents, is associated with lower educational quality and mental health. Grounded in the Study Demands–Resources (SD–R) and Conservation of Resources (COR) theories, this study investigates the relationship between school-based resources, family dynamics, and personal resources by examining how teacher emotional support is associated with burnout through family cohesion and meaning in life; (2) Methods: a moderated mediation model was tested using a sample of 1224 adolescents (Mage = 14.27, SD = 1.72; 48% female); (3) Results: Analysis revealed that: 1. Teacher emotional support significantly and negatively predicted student burnout (β = −0.28, *p* < 0.001). 2. Family cohesion partially mediated this relationship, accounting for 36% of the total effect. 3. Meaning in life significantly moderated both the direct path and the second half of the mediation pathway (family cohesion → burnout). Notably, meaning in life was associated with a stronger negative association between teacher emotional support and student burnout, but a weaker negative association between family cohesion and student burnout, a pattern consistent with differential resource utilization; (4) Conclusions: These findings suggest a differentiated pattern of resource interplay: school-based emotional resources may connect to family-based relational resources, and the protective role of each external resource may be further moderated by adolescents’ internal meaning systems. These findings highlight the agentic role of adolescents in resource management and point to the value of multi-system interventions.

## 1. Introduction

Adolescence is a critical period for the systematic acquisition of knowledge, skills, and social norms, where academic development serves as the central task. However, despite persistent efforts to reduce the academic burden in basic education, adolescents continue to experience significant academic pressure stemming from individual, familial, school, and societal factors. Surveys indicate that approximately 20% of adolescents report an increase in their academic load, rather than a decrease, while nearly two-thirds exhibit pronounced academic weariness (Ma & Cu, 2022). This highlights the prevalence of student burnout among adolescents. Student burnout is defined as a state of emotional, behavioral, and cognitive exhaustion arising from school-related stress, characterized by emotional exhaustion, depersonalization (or cynicism), and professional efficacy (Schaufeli et al., 2002). It is not only a common mental health issue but has also evolved into a novel form of dropout—termed ‘hidden’ or ‘psychological’ school dropout (Bilige & Gan, 2020; Li et al., 2021). This state fundamentally undermines learning motivation, thereby compromising educational quality and individual developmental potential. Therefore, examining how protective factors may help mitigate adolescent student burnout is crucial for fostering their positive adaptation and healthy development.

### 1.1. Theory and Hypotheses

The Study Demands–Resources (SD–R) theory provides a robust theoretical framework for understanding student burnout. It posits that the study environment consists of two distinct categories of characteristics: study demands and study resources (Bakker & Mostert, 2024; Salmela-Aro et al., 2022). Study demands refer to all the facets of studying that require sustained effort, that continuously consume physical and psychological energy (e.g., academic challenges and interpersonal conflicts). These demands are associated with a health impairment process, which can lead to emotional exhaustion, learning disengagement, and burnout (Bakker et al., 2023; Kaggwa et al., 2021). In contrast, study resources refer to aspects that are intrinsically motivating, capable of buffering stress, and fostering personal growth (e.g., social support and positive feedback). These resources are linked to a motivational process that fosters learning engagement, creativity, and well-being (Liu et al., 2023). These two types of characteristics not only operate independently but also interact (Lesener et al., 2020); specifically, study resources can buffer the detrimental effects of high study demands (the buffer hypothesis) (Naylor, 2022), while appropriately challenging study demands may amplify the positive effects of resources on engagement and performance (the boost hypothesis) (Hospel & Galand, 2016).

The COR theory further emphasizes that individuals are driven to acquire, retain, foster, and protect valued resources, and that resource loss or the threat of loss is central to understanding stress and burnout (Halbesleben et al., 2014; Hobfoll, 1989; Hobfoll et al., 2018). Resources can be categorized by source (contextual vs. personal) and stability (structural vs. volatile), including objects (conditions), constructive resources (personal traits), social support, and energy resources (ten Brummelhuis & Bakker, 2012). Among these, social support is particularly relevant to student burnout, as it can directly replenish resource reservoirs and compensate for other deficient resources (Hobfoll, 2002). For adolescents, the academic process often involves prolonged resource investment. Without sufficient replenishment, resource reservoirs may become depleted, which is associated with student burnout. Therefore, addressing burnout may require not only reducing demands but also emphasizing the development, mobilization, and replenishment of resources.

However, the SD–R model focuses primarily on individual students—their perceptions of study demands and resources. Its account of how resources across systems (e.g., school and family) can interact remains relatively underdeveloped, and more research on these cross-system dynamics is needed (Bakker & Mostert, 2024). COR theory, with its emphasis on resource caravans and crossover processes (Hobfoll et al., 2018), complements this gap; it suggests that resources tend to co-occur and travel together, meaning that the availability of resources in one domain (e.g., teacher support at school) may co-occur with the accrual of resources in another (e.g., cohesive family relationships). By integrating these perspectives, the present study moves beyond examining isolated protective factors and instead investigates a sequential pathway in which school-based emotional resources may connect to family-based relational resources, which in turn relate to lower burnout. This integrated model offers a framework for understanding cross-contextual patterns of resource co-occurrence that neither the SD–R model nor COR theory alone would fully explain. This research aims to provide a theoretical basis for practical interventions.

#### 1.1.1. Teacher Emotional Support

Social support refers to the psychological and material resources that individuals acquire from their social networks, including family, friends, school, and community (House et al., 1988; Taylor, 2012). According to the buffering model, social support may mitigate the negative impact of stress on mental health (S. Cohen & Wills, 1985) and has been associated with lower levels of burnout (Halbesleben, 2006). Based on the source, social support can be categorized into family, peer, and teacher support (French et al., 2018; Rueger et al., 2016). Among these, teachers serve as essential guides in adolescent development, and the care and support they provide are considered critical protective factors for students’ academic adaptation and psychological development (Emslander et al., 2025). A meta-analysis further indicates that teacher support exhibits a particularly strong negative predictive effect on student burnout compared to other sources of support (Kim et al., 2017). In terms of content, social support is broadly divided into emotional support and instrumental support. The former provides care, understanding, and encouragement, which helps alleviate emotional distress; the latter emphasizes practical assistance and information sharing, thereby enhancing self-efficacy (Jolly et al., 2021). Therefore, by integrating both source and content dimensions, teacher emotional support, defined as students’ perceptions of care, understanding, respect, and encouragement from teachers (Hamre & Pianta, 2005), may emerge as a core protective factor against student burnout. From the perspective of COR theory, teacher emotional support serves as a vital social support resource that may enrich students’ psychological resource reserves. When students perceive such support, they may be better able to sustain learning motivation and cope with stress, which could relate to lower levels of burnout. From the perspective of the integrated SD–R/COR framework, teacher emotional support constitutes a contextual resource within the school system that may serve as the starting point of a resource chain, setting the stage for potential resource linkages across systems. Based on this reasoning, we propose the following hypothesis (H1): Teacher emotional support has a negative predictive relationship with adolescent student burnout.

#### 1.1.2. The Role of Family Cohesion

Ecological systems theory emphasizes that the family, as the most immediate microsystem, provides essential resources crucial to adolescent development (Bronfenbrenner, 1977). High-quality parent–adolescent relationships emerge as a major protective factor against adolescents’ psychosomatic problems (Huang et al., 2023). Research has shown that a positive family climate contributes to adolescents’ psychological well-being and adaptive functioning (Fritz et al., 2018; Kurock et al., 2022; Yule et al., 2019). Family cohesion, which reflects a healthy family environment or functioning, refers to the quality of mutual support and care among family members, as well as the perceived degree of emotional connection (Olson et al., 1987). It has been demonstrated to be associated with high-quality social support, psychological regulation (Farrell & Barnes, 1993), and lower levels of negative emotions (e.g., stress, anxiety, and depression) (Bian et al., 2024), and has been further linked to adaptive development and lower levels of student burnout (Y. Wang et al., 2021). However, family functioning is not uniformly protective (Zhu et al., 2023). For example, parental interference has been linked to elevated burnout and depressive symptoms (Angelini et al., 2026). Moreover, in different cultural contexts, including Chinese culture, the family serves as a crucial support resource for children’s schooling (Barger et al., 2019). This makes family cohesion particularly salient as both a protective resource and a potential pathway linking school-based support to adolescent adjustment.

According to COR theory, resources tend to aggregate in caravans and travel across life domains through crossover processes (Hobfoll et al., 2018). We propose that teacher emotional support may positively relate to family cohesion through a process of resource spillover. First, the positive emotions and psychological resources (e.g., self-efficacy, optimism) associated with teacher emotional support may persist across contexts, which may be linked to more positive perceptions of family relationships and fewer conflict-ridden interactions. Second, the interpersonal skills and perspectives acquired through supportive teacher–student relationships (e.g., empathic listening, constructive self-expression) may be directly transferred to the home, which may enhance the quality of communication and emotional connection among family members. Together, these two mechanisms may improve the quality of family interactions and increase perceived family cohesion. In other words, the emotional security experienced at school may spill over into the home environment, which may be associated with a more supportive family climate. This argument is consistent with the broader literature on work–family enrichment, which demonstrates that resources generated in one domain can produce positive experiences (Affective Path) and outcomes (Instrumental Path) and transfer to another (Greenhaus & Powell, 2006). This is also consistent with emerging evidence that school-based support can extend to family relationships among adolescents (Thompson et al., 2018). However, direct evidence linking teacher emotional support to family cohesion remains limited, and further exploration is needed. Thus, we propose that family cohesion may not only serve as a protective resource on its own but may also act as a pathway linking teacher emotional support to student burnout. Accordingly, we propose Hypothesis 2 (H2): Family cohesion mediates the relationship between teacher emotional support and adolescent student burnout.

#### 1.1.3. The Role of Meaning in Life

Grounded in the SD–R and COR theoretical perspectives, individuals are not merely passive recipients of stress but also active resource builders, capable of engaging in forward-looking coping through the accumulation of key personal resources. Meaning in life represents one such crucial personal resource. It refers to the sense made of, and significance felt regarding, the nature of one’s being and existence (Steger et al., 2006). Frankl posited that the search for meaning is a fundamental human motive, and the absence of meaning lies at the root of psychological suffering and distress (Frankl, 1963). Empirical research confirms that meaning in life serves as a protective factor for mental health (King et al., 2016), and its deficiency constitutes a significant risk factor for student burnout (Hooker et al., 2020). Specifically, the perception of meaning in life may be associated with intrinsic learning desire and motivation among adolescent students, as well as a lower likelihood of student burnout (Güngör & Sari, 2022). According to the COR theory, meaning in life, as a key constructive resource, is associated with higher perceived social support, and individuals can reconstruct the sense of meaning through social connections during stressful periods (O’Donnell et al., 2014). Within the SD–R framework, meaning in life is an internal, belief-based personal resource that may compensate for insufficient external learning resources and moderate the negative association between high study demands on students (Bakker & Mostert, 2024). These perspectives suggest that adolescents with higher levels of meaning in life may be more adept at recognizing, accepting, and effectively utilizing social support from teachers and families, which could potentially enhance their overall resource reserves and resilience to academic stress. Therefore, the buffering effect of the care and support perceived by adolescents in both the school and family support systems on student burnout may be moderated by the sense of meaning in life. Notably, because meaning in life operates as an internal, belief-based personal resource, its moderating role is expected to manifest primarily in how external resources translate into individual outcomes rather than in how external resources interact with one another. Specifically, meaning in life may influence the extent to which adolescents benefit from available support, but it may not necessarily alter the degree to which school-based support spills over into the family. Based on this reasoning, we propose the following hypothesis 3 (H3): Meaning in life moderates the direct relationship between teacher emotional support and student burnout, as well as the second half of the mediation process.

**Figure 1:**
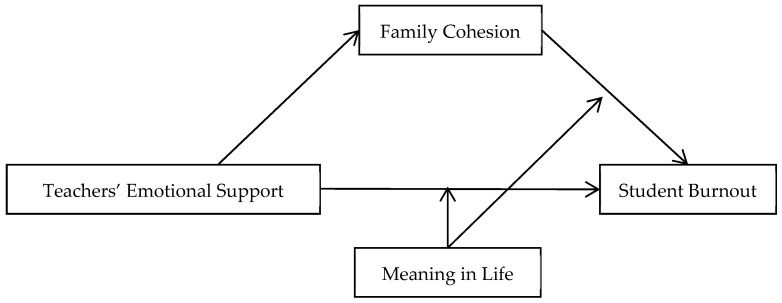
Moderated mediation model.

In summary, this study proposes and tests an integrated theoretical model (see Figure 1) to examine the hypothesized associations through which teacher emotional support relates to student burnout in adolescents. Specifically, the model examines the mediating role of family cohesion and the moderating role of meaning in life on both the direct path and the second half of the indirect path.

## 2. Materials and Methods

### 2.1. Participants

This study employed a convenience sampling method to survey students from six schools in Tianjin. Tianjin is a large, directly controlled municipality in northern China with a predominantly urban population. All students in grades 7–12 from the participating classes were invited. No exclusion criteria were imposed beyond consent requirements. After excluding questionnaires with repetitive responses or those that could not be matched, a total of 1224 valid questionnaires were included; the effective recovery rate is 99.5%. A total of 1230 students were initially approached; given the extremely high response rate, non-response bias is unlikely to be a concern. The participants ranged in age from 12 to 18 years (M = 14.27, SD = 1.72). In terms of grade distribution, 32.1% were in Grade 7, 21.8% in Grade 8, 6.9% in Grade 9, 19.7% in Grade 10, 13.3% in Grade 11, and 6.2% in Grade 12. Regarding gender, 52% of the participants were male, and 48% were female.

This study employed a cross-sectional, correlational design. This study was approved by the Ethics Review Committee of Tianjin Normal University (Ethics No.: XL20240909D). All procedures involving human participants were performed in accordance with the ethical standards of the institutional research committee and with the Declaration of Helsinki. The participants in this study were junior and senior high school students. Informed consent was obtained from both the students’ guardians and their schools before the data collection. The survey administration was supervised by psychology teachers and homeroom teachers. To reduce response bias, students were instructed to work independently without discussion, assured of anonymity, and told that there were no right or wrong answers. Data were collected on a per-class basis. The instructions clearly explained the purpose of the survey to the participants, and confidentiality was assured, with the assurance that data would be used solely for research purposes. During the administration, no participants reported difficulty in understanding the questionnaire items. Students were instructed not to communicate inappropriately and to comply with classroom discipline throughout the process.

### 2.2. Measures

#### 2.2.1. Adolescent Student Burnout Inventory

The Adolescent Student Burnout Scale, developed by Wu et al. (2010), was used. It contains 16 items and is structured around three dimensions: emotional exhaustion, academic alienation, and reduced sense of accomplishment. Responses were rated on a 5-point Likert scale (1 = very untrue of me, 5 = very true of me), with higher scores indicating a greater level of student burnout. In this study, the overall Cronbach’s α for the scale was 0.89, and the α coefficients for the subscales ranged from 0.78 to 0.85.

Confirmatory factor analysis (CFA) of the three-factor model yielded: χ^2^ = 904.660, *df* = 101, CFI = 0.903, TLI = 0.885, RMSEA = 0.081, SRMR = 0.060. Standardized factor loadings ranged from 0.42 to 0.87 (*ps* < 0.001), indicating that all items loaded substantially on their respective factors. Given the sensitivity of chi square to large sample size, other fit indices collectively support acceptable construct validity.

#### 2.2.2. Middle School Student Perceived Teachers’ Emotional Support Questionnaire

The Chinese version of the Teacher Emotional Support Questionnaire, developed by Gao et al. (2017), was used. This scale consists of 18 items and includes four dimensions: understanding students, caring for students, respecting students, and encouraging students. In this study, the overall Cronbach’s α for the scale was 0.96, and the α coefficients for the subscales ranged from 0.90 to 0.95.

Confirmatory factor analysis (CFA) of the four-factor model yielded: χ^2^ = 1541.747, *df* = 129, CFI = 0.929, TLI = 0.916, RMSEA = 0.095, SRMR = 0.054. Standardized factor loadings ranged from 0.50 to 0.94 (*ps* < 0.001), indicating that all items loaded substantially on their respective factors. Given the sensitivity of chi-square to large sample size, other fit indices collectively support acceptable construct validity.

#### 2.2.3. Family Adaptability and Cohesion Evaluation Scale (FACESII-CV)

The Family Cohesion subscale from the Chinese version of the Family Adaptation and Cohesion Evaluation Scales, modified by Fei et al. (1991), was used. This subscale consists of 16 items. Responses were recorded on a 5-point Likert scale (1 = never, 5 = always), with higher scores indicating a greater level of perceived family cohesion. In the present study, the Cronbach’s α for this subscale was 0.90.

Confirmatory factor analysis of the one-factor model yielded: χ^2^ = 1388.931, *df* = 104, CFI = 0.853, TLI = 0.830, RMSEA = 0.101, SRMR = 0.073. Although these fit indices were slightly below the recommended cutoffs, the scale showed good internal consistency.

#### 2.2.4. Meaning in Life Questionnaire

The adolescent version of the Meaning in Life Questionnaire, revised by X. Q. Wang (2013), was used. This scale consists of 10 items and encompasses two dimensions: the presence of meaning (MLQ-P) and the search for meaning (MLQ-S). Responses were measured on a seven-point Likert scale (1 = completely untrue; 7 = completely true), with higher scores reflecting a greater level of meaning in life. In this study, the overall Cronbach’s α for the scale was 0.84, and the coefficients for the two subscales ranged from 0.86 to 0.87.

Confirmatory factor analysis (CFA) of the two-factor model yielded: χ^2^ = 314.465, *df* = 34, CFI = 0.951, TLI = 0.935, RMSEA = 0.082, SRMR = 0.054. Standardized factor loadings ranged from 0.55 to 0.87 (*ps* < 0.001).

### 2.3. Statistical Analysis

We employed SPSS 26.0 for descriptive statistics, common method bias test, and correlation analysis, and used Mplus 8.3 to perform CFA on the questionnaire data. All main analyses were performed using observed composite scores, not latent variables. Mediation analysis and moderated mediation analysis were conducted using Model 4 and Model 15, respectively, of the PROCESS v4.2 macro for SPSS (Hayes, 2012), because the main research questions focus on linear relationships among manifest variables, and PROCESS does not accommodate latent variables. A bias-corrected bootstrap method with 5000 resamples was used to test the significance of the effects, with results reported using 95% confidence intervals.

## 3. Results

### 3.1. Preliminary Analyses

All data in this study were collected via self-report measures, which can introduce common method bias. To examine this potential bias, we conducted Harman’s single-factor test (Podsakoff et al., 2003). The results showed that ten factors with eigenvalues greater than 1 were extracted, and the first factor accounted for 28.55% of the total variance, which is well below the conventional critical threshold of 40%. This indicates that common method bias was not a significant concern in the current study.

All variables were mean-centered prior to analysis. Multicollinearity was tested in the full model including both interaction terms; all VIFs were below 2.0, indicating no multicollinearity. The Q-Q plot of residuals showed only minor tail deviations. Given the large sample size (*N* = 1224), OLS regression is robust to moderate non-normality. More importantly, all inferences were based on bootstrapping (5000 resamples), which does not assume normally distributed residuals. Therefore, the minor deviations in the Q-Q plot do not affect the validity of the conclusions.

### 3.2. Descriptive Statistics and Correlation Analysis

As shown in Table 1, Pearson correlation analyses revealed that teacher emotional support was significantly negatively correlated with student burnout (*r* = −0.43, *p* < 0.001) and significantly positively correlated with both meaning in life and family cohesion (r = 0.40 and 0.42, respectively, *ps* < 0.001). Student burnout was significantly negatively correlated with meaning in life and family cohesion (*r* = −0.45 and −0.48, respectively, *ps* < 0.001). A significant positive correlation was also observed between meaning in life and family cohesion (*r* = 0.43, *p* < 0.001). These significant intercorrelations among the four key variables support further moderated mediation analysis.

### 3.3. The Mediating Role of Family Cohesion

Following J. Cohen’s (1988) guidelines, standardized coefficients (β) of approximately 0.1, 0.3, and 0.5 were interpreted as small, medium, and large effects, respectively. Using Model 4 from the PROCESS macro for SPSS, we examined the mediating role of family cohesion in the relationship between teacher emotional support and adolescent student burnout, while controlling for gender and grade. The results (see Table 2) showed that teacher emotional support significantly and positively predicted family cohesion (β = 0.43, *t* = 16.57, *p* < 0.001) and significantly and negatively predicted student burnout (β = −0.28, *t* = −10.35, *p* < 0.001). Family cohesion also significantly and negatively predicted student burnout (β = −0.36, *t* = −13.56, *p* < 0.001). Further analysis using the bias-corrected bootstrap method (see Table 3) revealed a significant direct effect of teacher emotional support on student burnout (effect = −0.245, 95% CI [−0.29, −0.20]). The indirect effect of teacher emotional support on student burnout through family cohesion was also significant (effect = −0.138, 95% CI [−0.17, −0.11]). Because the confidence interval for the indirect effect did not include zero, these results indicate that family cohesion partially mediates the relationship between teacher emotional support and student burnout. According to J. Cohen’s (1988) guidelines, these standardized coefficients represent small to moderate effect sizes.

### 3.4. Moderated Mediation Effect Test

Using Model 15 of the PROCESS macro for SPSS, we tested the moderated mediation model while controlling for gender and grade. The results (see Table 4) showed that the interaction term between teacher emotional support and meaning in life significantly predicted student burnout (β = −0.05, *p* < 0.05). Similarly, the interaction between family cohesion and meaning in life also significantly predicted student burnout (β = 0.06, *p* < 0.01). These findings indicate that meaning in life moderates both the relationship between teacher emotional support and student burnout and the relationship between family cohesion and student burnout.

**Figure 2:**
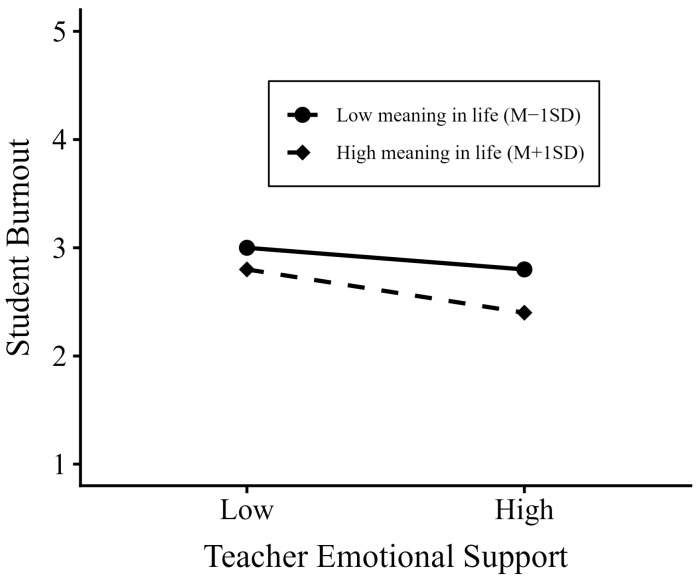
The Moderating Effect of Meaning in Life on the Relationship Between Teacher Emotional Support and Student Burnout.

**Figure 3:**
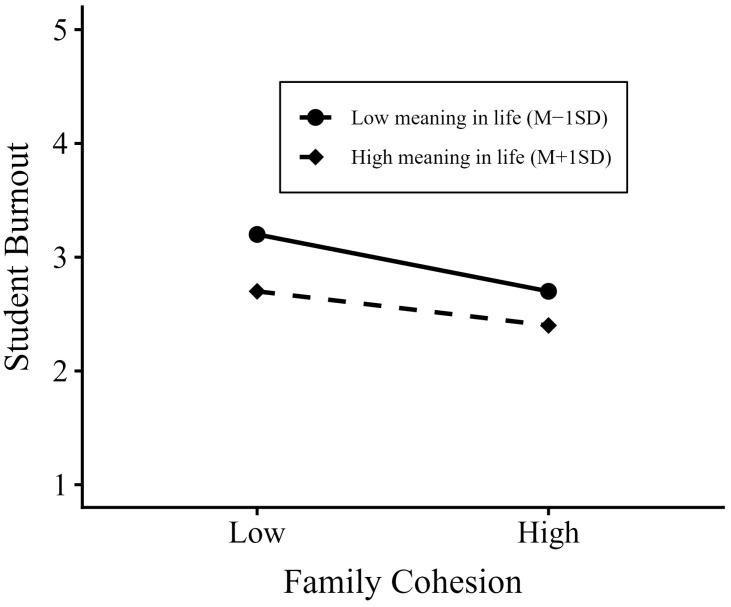
The Moderating Effect of Meaning in Life on the Relationship Between Family Cohesion and Student Burnout.

To further illustrate the moderating role of meaning in life, simple slope analyses were conducted at one standard deviation above and below the mean of meaning in life (see Figure 2 and Figure 3). For the teacher emotional support → student burnout path (Figure 2), the negative connection was stronger at high meaning (+1SD: β = −0.25, *t* = −7.34, *p* < 0.001) than at low meaning (−1SD: β = −0.14, *t* = −4.48, *p* < 0.001), indicating that meaning in life amplified this protective effect. For the family cohesion → student burnout path (Figure 3), the negative effect was stronger at low meaning (−1SD: β = −0.31, *t* = −9.69, *p* < 0.001) than at high meaning (+1SD: β = −0.19, *t* = −5.34, *p* < 0.001), indicating that meaning in life attenuated this protective connection.

## 4. Discussion

This study extends the Study Demands–Resources (SD–R) theory, originally developed within higher education contexts (Bakker & Mostert, 2024), to the basic education stage, thereby supporting its explanatory power among adolescents. By integrating the SD–R theory with COR theory, we constructed a moderated mediation model to systematically investigate the pathways through which teacher emotional support is associated with adolescent student burnout. The results not only confirmed the direct negative predictive effect of teacher emotional support on student burnout but also revealed the mediating role of family cohesion and the moderating role of meaning in life on both the direct path and the second half of the indirect path (family cohesion → student burnout). These findings provide an integrated multi-system perspective for understanding the conditional associations related to adolescent student burnout.

### 4.1. The Direct Effect of Teacher Emotional Support on Student Burnout

The findings of this study reveal that teacher emotional support is significantly and negatively associated with adolescent student burnout (β = −0.28, *p* < 0.001), thereby confirming Hypothesis 1. This finding is consistent with previous research (Romano et al., 2020; Sun et al., 2025). In accordance with both SD-R and COR theories (Hobfoll et al., 2018; Salmela-Aro et al., 2022), resources may serve as pivotal protective factors that help individuals cope with stress and prevent psychological depletion. When students perceive emotional care, understanding, and encouragement from their teachers, they may develop a sense of psychological safety, which could enable them to respond more positively to academic stressors and setbacks (Kidger et al., 2012). Thus, as key influencers within the school ecosystem, teachers may, through emotional support, help alleviate negative emotions triggered by academic pressure and bolster students’ psychological resources, which may in turn relate to lower levels of burnout. Moreover, this result further supports the unique role of teacher support relative to other sources of social support (Kim et al., 2017). Compared to family and peer support, teacher emotional support may be more directly intertwined with students’ academic tasks and self-efficacy (Evans et al., 2024). Such targeted encouragement and validation may buffer the frustration stemming from academic failure, thereby exerting a more specific mitigating association with the core symptoms of student burnout.

### 4.2. The Mediating Role of Family Cohesion

This study further showed that teacher emotional support is associated with lower student burnout not only directly but also indirectly through perceived family cohesion, thereby supporting Hypothesis 2. First, family cohesion was negatively associated with student burnout, which is consistent with prior research (Y. Wang et al., 2021; Yu et al., 2021). According to ecological systems theory, individual development is embedded within interacting environmental systems characterized by continuous person–environment transactions (Bronfenbrenner, 1977). As a primary microsystem, higher family cohesion may provide a secure and supportive emotional base, which could buffer the psychological depletion arising from academic pressure and reduce the likelihood of burnout.

Second, mediation analysis indicated that family cohesion partially mediates the relationship between teacher emotional support and student burnout (indirect effect = −0.14, accounting for 36% of the total effect). This finding is supported by the integrated SD–R/COR framework, which suggests that resources from different systems can be integrated to foster positive outcomes (Bakker et al., 2023; Bakker & Mostert, 2024). It lends support to the resource spillover hypothesis proposed in the introduction. However, because the specific processes, such as the affective transfer of positive emotions or the instrumental transfer of interpersonal skills, were not directly examined in this study, these interpretations should be treated as theoretically informed speculations rather than direct empirical conclusions. Alternative explanations are equally plausible. For example, teacher emotional support and family cohesion may both reflect a broader, unmeasured ecological context (e.g., overall school–family partnership quality or community socioeconomic resources) that is associated with lower burnout. The specific processes through which teacher support relates to family cohesion warrant direct investigation in future research.

Moreover, we acknowledge that the relationship between teacher support and family cohesion may be bidirectional. However, the present study prioritizes the teacher-to-family direction for two reasons. First, the integrated SD–R/COR framework adopted in this study positions school-based resources as the starting point of the hypothesized resource chain. Second, adolescence is a developmental period characterized by increasing time spent in school contexts and growing reliance on non-parental adult relationships (Eccles & Roeser, 2011), making the examination of how school-based resources shape family experiences particularly timely. Future longitudinal and experimental research is needed to disentangle the reciprocal dynamics between these constructs.

### 4.3. The Moderating Role of Meaning in Life

This study further showed that meaning in life moderated both the direct path and the latter stage of the indirect pathway (i.e., family cohesion → student burnout), thereby supporting Hypothesis 3. Notably, the direction of moderation differed between the two paths, a finding that is open to multiple interpretations and may be tentatively interpreted through the lens of resource functional properties of resources and the initiative of individual resource invocation.

For the direct path, meaning in life was associated with a stronger negative association between teacher emotional support and student burnout. This finding is consistent with the SD-R perspective on the synergy between personal and external resources (Bakker & Mostert, 2024; Jagodics & Szabó, 2023). As a stable, internal constructive resource, meaning in life may be associated with enhanced perception and utilization of exogenous support. One possible interpretation is that students with higher levels of meaning in life are more likely to interpret teacher care, understanding, and encouragement as positive affirmations of their academic value and potential. This synergy may relate to stronger learning motivation and less academic stress, which could, in turn, be associated with lower burnout. However, because these cognitive and affective processes were not directly measured, this interpretation remains speculative. Conversely, for the second stage of the mediation (family cohesion → burnout), higher levels of meaning in life were unexpectedly associated with a weaker negative association between family cohesion and student burnout. This result may be tentatively interpreted through COR theory’s resource investment principle and the resource gain spiral (Halbesleben et al., 2014; Hobfoll et al., 2018). This perspective suggests that individuals tend to invest existing resources to acquire, retain, and protect other valued resources. A high level of meaning in life itself may constitute a robust internal resource system that associated with intrinsic value affirmation and emotional regulation. One possible interpretation is that, when confronting specific academic challenges, individuals with substantial internal resources may rely more heavily on external supports that are closely aligned with the academic context—such as teacher emotional support. In contrast, although family cohesion provides important emotional security, its perceived utility in addressing specific academic demands may be less pronounced when individuals already possess a strong internal meaning-making system. This does not imply that family support is irrelevant; it is plausible that when individuals possess sufficient internal resources, their observed reliance on specific external supports with overlapping roles is reduced, which may be associated with a diminished buffering effect of family cohesion in the model.

In summary, the moderating role of meaning in life illustrates the complex interplay among multi-system resources. Although these differential moderation patterns could be theoretically interpretable, they should be treated with caution. Alternative explanations, including the possibility that unmeasured third variables (e.g., overall family socioeconomic status or school climate quality) drive the observed interactions, cannot be ruled out in the present cross-sectional design. Future research incorporating experimental or longitudinal designs, as well as multi-informant assessments, is needed to adjudicate among these competing interpretations.

### 4.4. Limitations

Although this study provides preliminary insights into how teacher emotional support is associated with student burnout through family cohesion, along with the moderating role of meaning in life, several limitations should be acknowledged. First, the cross-sectional design precludes establishing definitive causal inferences among the variables. Therefore, future research should employ longitudinal or experimental designs to better capture the dynamic processes underlying the proposed pathways. Second, the use of convenience sampling (students from six schools in Tianjin) limits the generalizability of the findings. Although our sample covered a wide age range and grade levels, caution is needed when extrapolating the results to other regions, educational systems, or populations. Future studies with probability sampling methods are required to enhance external validity.

Third, all variables were assessed via self-report from a single informant, which increases the risk of inflated associations due to shared method variance and measurement overlap among constructs. Although Harman’s single-factor test suggested no severe common method variance, this approach is widely regarded as insufficient for definitively ruling out method effects. Although we have employed various methods to try to rule out this possibility, it may remain insufficient. Future research should incorporate multi-informant measures, such as reports from teachers and parents, to reduce shared method variance and enhance the robustness of the findings. Fourth, the measurement model for family cohesion exhibited suboptimal fit indices, raising concerns about construct validity in this sample. Although the scale was retained based on its established theoretical relevance, the findings involving family cohesion should be interpreted with caution. Future studies would benefit from using alternative or refined measures of family cohesion that demonstrate stronger psychometric properties in adolescent samples.

Despite these limitations, the present findings offer several implications for educational practice. First, the significant mediating role of family cohesion suggests that interventions aimed at reducing student burnout may benefit from simultaneously strengthening both school-based and family-based support systems. For example, schools could implement programs that foster teacher emotional support while also engaging parents in activities that promote cohesive family interactions. Second, the moderating role of meaning in life highlights the potential value of meaning-oriented interventions, such as structured life-crafting exercises or purpose clarification activities. Thus, integrating these approaches into a multi-component intervention, combining teacher support enhancement, family relationship facilitation, and meaning-in-life cultivation, may offer a promising strategy for alleviating student burnout. Future research is encouraged to test the practical efficacy of such multi-faceted intervention programs using rigorous experimental or quasi-experimental designs.

## 5. Conclusions

The present study examined how teacher emotional support is associated with adolescent student burnout through a moderated mediation model. Taken together, these findings point to a differentiated pattern of resource interplay: school-based emotional resources may connect to family-based relational resources in a sequential chain, while the protective role of each external resource appears to be further shaped by adolescents’ internal meaning systems. These results highlight a complex, multi-systemic mechanism of resource mobilization, underscoring the importance of integrating school-based support, family dynamics, and internal psychological resources to help reduce adolescent student burnout.

## Figures and Tables

**Table 1:** Descriptive Statistics and Correlations.

	*M*	*SD*	Gender	Grade	Teachers’ Emotional Support	Student Burnout	Meaning in Life
Gender	1.52	0.50	1				
Grade	8.70	3.49	0.03	1			
Teachers’ Emotional Support	62.82	30.33	−0.02	0.01	1		
Student Burnout	37.51	31.47	0.02	−0.01	−0.43 ***	1	
Meaning in Life	44.51	26.58	0.02	0.12 **	0.40 ***	−0.45 ***	1
Family Cohesion	50.63	33.94	0.03	−0.01	0.42 ***	−0.48 ***	0.43 ***

Note: ** *p* < 0.01, *** *p* < 0.001.

**Table 2:** Regression Analysis of Family Cohesion and Student Burnout.

	Family Cohesion		Student Burnout	
	β	*t*	β	*t*
Gender	−0.02	−0.61	0.08 **	3.20
Grade	0.05 *	1.97	0.06 **	2.60
Teachers’ Emotional Support	0.43 ***	16.57	−0.28 ***	−10.35
Family Cohesion			−0.36 ***	−13.56
R^2^	0.19		0.30	
*F*	91.92 ***		131.34 ***	

Note: * *p* < 0.05, ** *p* < 0.01, *** *p* < 0.001.

**Table 3:** Mediating effect analysis.

Type of Effect	Effect	BootSE	BootLLCI	BootULCI	Proportion of Total Effect
Total Effect	−0.383	0.02	−0.43	−0.34	
Direct Effect	−0.245	0.02	−0.29	−0.20	64%
Indirect Effect	−0.138	0.01	−0.17	−0.11	36%

**Table 4:** Testing of the moderated mediation model.

Variable	Family Cohesion		Student Burnout	
	β	*t*	β	*t*
Gender	−0.03	−0.61	0.11 **	3.22
Grade	0.08 *	1.97	0.10 **	2.74
Teachers’ Emotional Support	0.42 ***	16.57	−0.19 ***	−8.11
Family Cohesion			−0.25 ***	−10.19
Meaning in Life			−0.36 ***	−4.50
Teachers’ Emotional Support × Meaning in Life			−0.05 *	−2.38
Family Cohesion × Meaning in Life			0.06 **	2.58
R^2^	0.19		0.35	
*F*	91.92 ***		92.82 ***	

Note: * *p* < 0.05, ** *p* < 0.01, *** *p* < 0.001.

## Data Availability

The data presented in this study are available upon request from the corresponding author.
